# Much ado about nothing – a decade of porous materials research

**DOI:** 10.1038/s41467-020-18746-5

**Published:** 2020-10-02

**Authors:** Arne Thomas

**Affiliations:** Department of Chemistry, Functional Materials, Technische Universität Berlin, Hardenbergstraße 40, 10623 Berlin, Germany

**Keywords:** Porous materials, Two-dimensional materials

## Abstract

Research on porous materials has produced intriguing novel materials in terms of composition, porosity and structures recently. This perspective aims to provide a short overview on some of the highlights reported within the last decade in this field.

Porous materials research (thus actually the science about the creation of nothing) has fascinated material chemists since decades. The possibility to create smaller and smaller holes and to control their arrangement has led to a range of new materials which are now used in everyday or industrial applications. Reviews on porous materials normally start with ancient usages of activated charcoals (a long time ago) or at least the discovery of the first natural or synthetic zeolites (nearly 250 and 70 years ago, respectively). This short perspective will however just describe some highlights of the recent 10 years of research on porous materials—and this certainly in a highly subjective manner. But is it actually worth to make such an effort? Did anything exciting happened in the field or is this one of these research areas, where progress can just be proven by an increasing number of publications but the last essential break-through has been achieved decades ago? As a measure, we can probably state that real progress has just then be achieved, when at this day things have been accomplished, which would have not been imaginable 10 years ago. Fortunately, everyone working on porous materials will be able to name several of such examples without much thinking and the author’s first thoughts are described below. Other colleagues would certainly add numerous further examples when thinking of the last 10 years breakthroughs in the field of porous materials.

## Porosity and surface area

Researchers working on porous materials are actually obsessed by the “nothing”, which can be introduced into a material. This value is expressed as porosity, namely the pore volume and the related created surface area. It doesn’t matter if you talk to specialists in zeolites, carbons, mesoporous oxides, metal-organic frameworks (MOFs) or porous polymers, in these terms they all speak the same language.

**Fig. 1:**
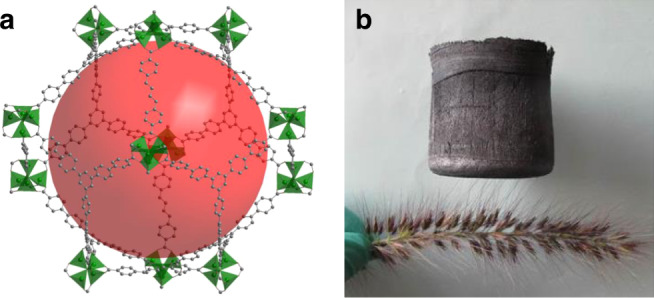
High and low. Materials with the **a** highest surface area (DUT-60)^1^ and **b** lowest density (ultra‐flyweight aerogels (UFAs))^2^ reported so far. The figure content is reproduced with permission from Wiley VCH^2^.

The specific surface area is the area of a material accessible to gas molecules in the dry state and can be largely increased when pores are introduced into a material. In 2010, MOFs with surface areas exceeding 6000 m^2^/g were firstly reported, 2012 the 7000 m^2^/g mark was surpassed and this came to a preliminary end with a MOF exhibiting the current world record of 7836 m^2^/g in 2018 (Fig. 1a)^1^. This is approximately the surface area of a football field you can hold in your hand! This came with a pore volume of 5.02 cm^3^/g, i.e., 90.3% of the material is free volume and a density of just 0.187 g/cm^3^. But these MOFs are still heavyweights compared to another material class which has gained momentum in the last decade. Indeed, more “nothing” can be introduced, when the pores are getting larger, reducing the amount of left-over material to a minimum, as it is seen in new generations of aerogels, e.g., graphene aerogels with a porosity of ~99.9% and a density down to 0.00016 g/cm^3^ ^2^ (Fig. 1b). Aerogels are impressive also when they are made from heavy materials, thus very recently a gold aerogel was reported with a density of just 0.006 g/cm^3^, which is 0.03 % of the density of bulk gold^3^.

## Composition and connectivity

While the world of porous, high surface area materials was 10 years ago mainly divided up among inorganic (zeolites, ordered mesoporous oxides, porous carbons) and organic–inorganic materials (MOFs), purely organic materials, like covalent organic frameworks (COFs) and conjugated microporous polymers (CMPs) have now also entered the stage. While the first COFs and CMPs were reported already in the 2010s they have experienced tremendous interest within the last 10 years. This can be explained by their unusual properties originating from their entirely organic, often fully π-conjugated composition. The first metal-free photocatalyst for hydrogen generation from water, a polymeric carbon nitride, was reported in 2009, but it took another few years until it was realized that also COFs and CMPs could be used for this application^4,5^. Since then, photocatalysis became one major application investigated for organic porous materials. Another research focus within the last 10 years was to find new reactions and linkages to connect organic molecules into high surface area materials. For highly crystalline COFs it was generally accepted that they can just be formed from chemically weak covalent bonds, as reversible covalent bond formations are required. Indeed, beginning of this decade the only known highly crystalline COFs were formed by boron–oxygen and later imine bounds, as these can be reopened easily by hydrolysis. Within the last 10 years however, the number of possible COF linkages have largely expanded and nowadays they can be as well formed from chemically stable covalent bonds, which culminated in the formation of entirely sp^2^-carbon connected frameworks^6^. While this expansion of stability of COFs on the one hand is a milestone for crystalline organic porous materials, also other connectivities have been investigated to form high surface area organic materials. Thus porous materials from organic building blocks with high permanent surface areas have been built via hydrogen bonds (HOFs)^7^, supramolecular interactions (SOFs)^8^ or hypercoordinate main group elements (SiCOFs)^9^. A highly unusual bonding pattern was finally reported within a COFs showing a mechanical interlocked architecture with fabric-like woven organic threads^10^.

## Dimensions and dynamics

**Fig. 2:**
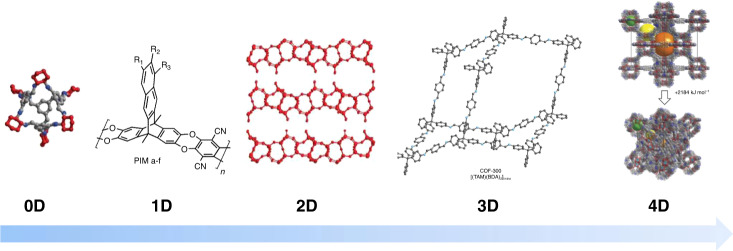
Dimensions of porous materials. From left to right: Examples of 0D nanocages^11^, 1D polymers of intrinsic microporosity^12^, 2D zeolite layers^14^, 3D COFs^15^ and a 4D MOF changing its structure during pressure increase^19^. The figure content is reproduced with permission from Nature/Springer/Palgrave^11,14,19^, the Royal Society of Chemistry^12^ and AAAS^15^.

Ten years ago, most porous materials were 3D materials, i.e., the material and its porosity expanded into all three dimensions. Sure there were a few exceptions, like the 1D polymers of intrinsic microporosity (PIMs) and 2D layered COFs, but zeolites, porous carbons and most MOFs all felt into this category. This has changed within the last 10 years (Fig. 2). An intriguing achievement was the development of discrete molecules with permanent porosity, called organic cage compounds, which thus can be described as 0D porous materials. These porous cages are soluble, highlighted in the development of a porous liquid, thus a concentrated solution of cage compounds whose solvent molecules are too large to enter the pores and which can thus take up a high amount of gas molecules^11^. But also the mentioned 1D PIMs have seen remarkable progress and within the last 10 years membranes composed of new PIMs have redefined the Robeson upper bound several times, a benchmark for gas pair separations (e.g., CH_4_/CO_2_ or CO_2_/N_2_) in membranes^12^. MOFs on the other hand went 2D. While exfoliation of layered MOFs was known since some time, this field has gained further momentum by the development of π-conjugated 2D MOFs prepared by bottom-up approaches, especially due to their astonishing high electric conductivity^13^. New dimensions were also exploited for zeolites with the generation of 2D zeolite nanosheets. Intriguingly, the layers could be used to reassemble 3D zeolite structures, which can hardly be produced by direct synthesis routes^14^. While the latter materials have started as 3D compounds whose dimensionality were reduced over time, the original 2D COFs have been expanded to 3D COFs over the years, culminating in the formation of single crystals of these entirely covalently linked frameworks^15^, yielding a fascinating insight into the structure of such materials. Furthermore, a reversible 2D to 3D transformation has been recently observed in COFs, triggered by a light induced [2 + 2] cycloaddition between vinylene groups in separate COF layers^16,17^. But why stopping at the dimensions of our experiential reality? The timescale has been recognized as a new perspective to investigate the dynamics and spatiotemporal evolution, thus of 4D porous materials. It has been recognized that many porous materials are far from static, but can change their structure and porosity as result of an external stimuli, yielding to extraordinary properties such as selective recognition of small molecules^18^ or the quite counterintuitive negative gas adsorption, i.e., the spontaneous desorption of gas molecules occurring during pressure increase^19^.

## Beyond synthesis

It’s a common phrase nowadays that a material has been created by design. Even though many porous materials have indeed gained a structural precision and predictable function, that they seemed to be (and probably are) designed on a drawing board, every one of us know that real life in the laboratory still means to carry out countless reactions until a new material can be presented. Thus what´s ending up with a few lines in the experimental part of a publication is often the labor of weeks, months or even years. But there is hope—indeed the synthesis of porous materials (and synthesis in general) will probably look very different end of the next decade. Machine learning, big data and robotics have entered the field of porous material development and have already shown to yield materials, optimized in terms of structure, porosity and/or applications^20,21^. It is conceivable that this trend will revolutionize the way we do ((porous) materials) synthesis in the future. Thus the next ten years of porous materials research promise to be even more exaggerating as the last ones—fingers crossed.

## References

[1] Hönicke IM (2018). Balancing mechanical stability and ultrahigh porosity in crystalline framework. Mater. Angew. Chem. Int. Ed..

[2] Sun H, Xu Z, Gao C (2013). Multifunctional, ultra-flyweight, synergistically assembled carbon aerogels. Adv. Mater..

[3] Qian F (2020). Gold aerogel monoliths with tunable ultralow densities. Nano Lett..

[4] Kailasam K (2013). Room temperature synthesis of heptazine-based microporous polymer networks as photocatalysts for hydrogen evolution. Macromol. Rapid Commun..

[5] Stegbauer L, Schwinghammer K, Lotsch BV (2014). A hydrazone-based covalent organic framework for photocatalytic hydrogen production. Chem. Sci..

[6] Lyu H, Diercks CS, Zhu C, Yaghi OM (2019). Porous crystalline olefin-linked covalent organic frameworks. J. Am. Chem. Soc..

[7] He Y, Xiang S, Chen B (2011). A microporous hydrogen-bonded organic framework for highly selective C_2_H_2_/C_2_H_4_ separation at ambient temperature. J. Am. Chem. Soc..

[8] Zhang K-D (2013). Toward a single-layer two-dimensional honeycomb supramolecular organic framework in water. J. Am. Chem. Soc..

[9] Roeser J (2017). Anionic silicate organic frameworks constructed from hexacoordinate silicon centres. Nat. Chem..

[10] Liu Y (2016). Weaving of organic threads into a crystalline covalent organic framework. Science.

[11] Giri N (2015). Liquids with permanent porosity. Nature.

[12] Comesana-Gandara B (2019). Redefining the Robeson upper bounds for CO_2_/CH_4_ and CO_2_/N-2 separations using a series of ultrapermeable benzotriptycene-based polymers of intrinsic microporosity. Energy Environ. Sci..

[13] Sun L, Campbell MG, Dinca M (2016). Electrically conductive porous metal-organic frameworks. Angew. Chem. Int. Ed..

[14] Roth WJ (2013). A family of zeolites with controlled pore size prepared using a top-down method. Nat. Chem..

[15] Ma T (2018). Single-crystal X-ray diffraction structures of covalent organic frameworks. Science.

[16] Acharjya A, Pachfule P, Roeser J, Schmitt F-J, Thomas A (2019). Vinylene-linked covalent organic frameworks by base-catalyzed aldol condensation. Angew. Chem. Int. Ed..

[17] Jadhav T (2020). Transformation between 2D and 3D covalent organic frameworks via reversible [2+2] cycloaddition. J. Am. Chem. Soc..

[18] Sato H (2014). Self-accelerating CO sorption in a soft nanoporous crystal. Science.

[19] Krause S (2016). A pressure-amplifying framework material with negative gas adsorption transitions. Nature.

[20] Burger B (2020). A mobile robotic chemist. Nature.

[21] Moosavi SM (2019). Capturing chemical intuition in synthesis of metal-organic frameworks. Nat. Commun..

